# Intra-mitochondrial biomineralization for inducing apoptosis of cancer cells†
†Electronic supplementary information (ESI) available. See DOI: 10.1039/c7sc05189a


**DOI:** 10.1039/c7sc05189a

**Published:** 2018-01-25

**Authors:** Sangpil Kim, L. Palanikumar, Huyeon Choi, M. T. Jeena, Chaekyu Kim, Ja-Hyoung Ryu

**Affiliations:** a Department of Chemistry , School of Natural Sciences , Ulsan National Institute of Science and Technology (UNIST) , Ulsan 44919 , Republic of Korea . Email: chaekyu@unist.ac.kr ; Email: jhryu@unist.ac.kr

## Abstract

The use of biomineralization that regulates cellular functions has emerged as a potential therapeutic tool. However, the lack of selectivity still limits its therapeutic efficacy. Here, we report a subcellular-targeting biomineralization system featuring a triphenylphosphonium cation (TPP) (the mitochondria-targeting moiety) and trialkoxysilane (the biomineralization moiety *via* silicification). The TPP-containing trialkoxysilane exhibited approximately seven times greater cellular uptake into cancer cells (SCC7) than into normal cells (HEK293T) due to the more negative mitochondrial membrane potentials of the cancer cells. In turn, its accumulation inside mitochondria (pH 8) induces specific silicification, leading to the formation of silica particles in the mitochondrial matrix and further activation of apoptosis. *In vivo* assessment confirmed that the biomineralization system efficiently inhibits tumor growth in a mouse xenograft cancer model. Exploiting both the subcellular specificity and the targeting strategy provides new insight into the use of intracellular biomineralization for targeted cancer therapy.

## Abstract 2

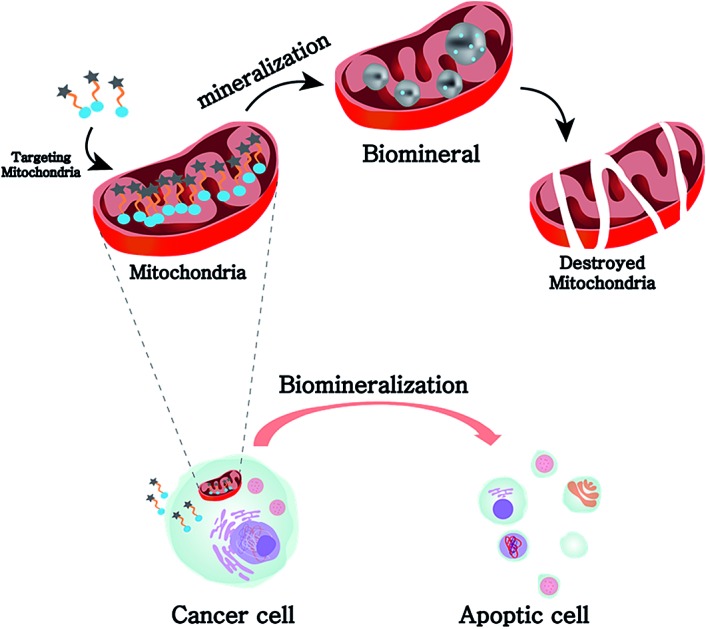
Mitochondria targeting mineralization can form biominerals inside cancerous mitochondria through concentration dependent silicification, resulting in dysfunction of mitochondria leading to apoptosis. These results suggest potential therapeutics for cancer treatment.

## Introduction

Biomineralization is an essential physiological process, which efficiently utilizes inorganic elements that are available in body fluids.^1^ It involves homeostasis of bones and skeletal tissues for suitable mechanical functions and internal supports through the cell-mediated deposition and resorption of inorganic substances.^2^ In addition, sequestration and secretion of intracellular mineral deposits (*e.g.* amorphous calcium phosphate) contribute to the regulation of cellular ion homeostasis, fate, and signaling roles.^3^,^4^ Generally, these biomineralization processes are tightly regulated by modular genetic mechanisms and physiological processes. Therefore, a pharmacological approach for regulating biomineralization could provide a new tool for controlling cellular functions and would have potential therapeutic applications.

Recently, the potential use of extracellular biomineralization (*e.g.* calcification and silicification) *in vitro* and *in vivo* has been demonstrated in cell and cancer therapies.^5^–^8^ However, abnormal extracellular mineral deposits can cause cellular damage to normal cells near the mineral nucleation sites, diffusion barriers for biological molecules, and the loss of cellular susceptibility to environmental changes.^5^ In this regard, incorporating intracellular targeting strategies into the biomineralization system would be beneficial due to the targeted delivery capability for desired therapeutics, reducing side effects.^9^ A number of particular subcellular compartments including the endosome, endoplasmic reticulum, Golgi complex, mitochondria, and nucleus have been studied for enhancing anticancer efficiency.^10^

Of these organelles, the targeting of mitochondria has offered a promising strategy to improve chemotherapy efficiency by reducing toxic side effects.^11^–^13^ Specifically, conjugation of the drug to triphenylphosphonium (TPP), a lipophilic cation, enables more than ∼10 times greater accumulation of the drug in the mitochondria of cancer cells than in those of normal cells as the mitochondrial membrane potential of cancer cells (∼–220 mV) is more negative than that of normal cells (∼–160 mV).^14^,^15^ The conjugation of TPP with bioactive molecules (*e.g.* small molecules and peptides) thus would be a promising approach to target and disrupt the mitochondria of cancer cells, enhancing the efficacy of cancer chemotherapy.^16^–^18^ Recently, we reported that the supramolecular polymerization of dipeptides inside the mitochondria induced the dysfunction of mitochondria by disrupting the membrane, resulting in the selective apoptosis of cancer cells. Due to the more negative mitochondrial membrane potentials in cancer cells compared to in normal cells, the TPP-conjugated molecules highly accumulated in the cancer cells and induced the self-assembled structures.^17^ Based on these previous reports and our observation, we hypothesized that biomineralization inside the mitochondria also would induce physical damage to the mitochondria and have a selective anti-cancer effect.

**Scheme 1:**
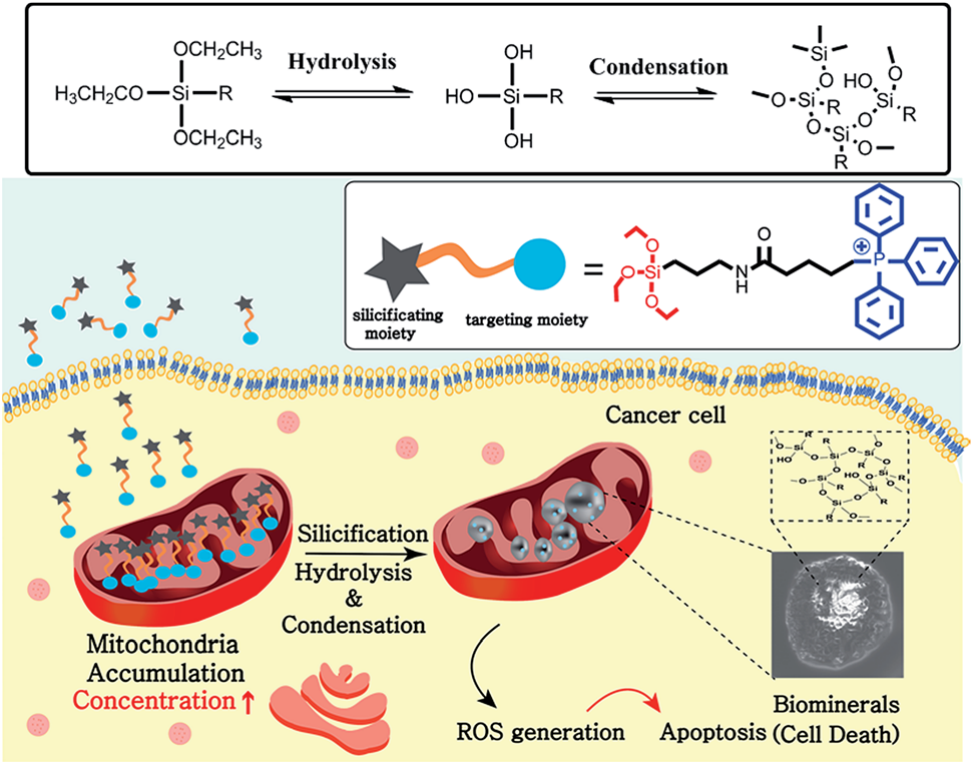
Representation of the selective accumulation of trialkoxysilane–TPP in cancerous mitochondria. This leads to mitochondria-specific silicification to form biominerals that induce cellular apoptosis.

Here, we report a mitochondria-targeting biomineralization system featuring TPP-conjugated trialkoxysilanes. The use of the trialkoxysilane takes advantage of environment-responsive silicification specific to the basic conditions of the mitochondrial matrix (∼pH 8). It induced the formation of silica particles that could act as mitochondrial dysfunction agents. Additionally, owing to the mitochondria targeting ability of TPP, the TPP-conjugated trialkoxysilanes exhibited preferential accumulation in the cancer cells over the normal cells. The silica particles depolarized and disrupted the mitochondrial membrane, generating reactive oxygen species (ROS). This further induced the dysfunction of mitochondria and activated apoptotic pathways, resulting in the anti-tumor effect *in vitro* and *in vivo*. These results showed that intra-mitochondrial biomineralization could provide a new tool for regulating cellular functions and therefore could have potential therapeutic applications (Scheme 1).

## Results and discussion

**Fig. 1:**
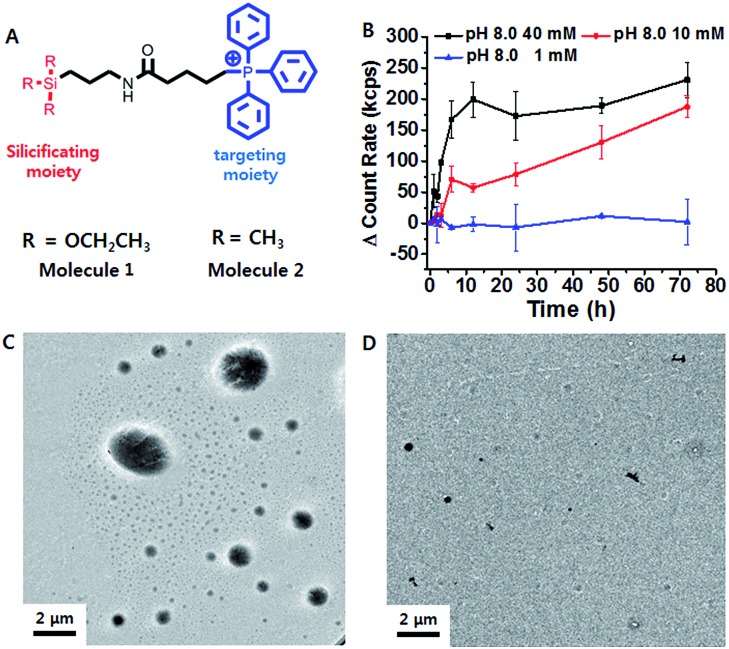
(A) Structure design of the mitochondria targeting molecule **1**, and the control molecule, **2**. (B) Time-dependent light scattering intensity of **1** (1, 10 and 40 mM) in ammonium buffer for 72 h. TEM images showing particle formation in (C) 40 mM and (D) 1 mM **1**.

Triethoxypropylsilane–TPP (**1**) was synthesized *via* a dicyclohexylcarbodiimide (DCC) coupling reaction and purified by size exclusion chromatography (ESI Scheme S1†). We hypothesized that the concentration of the trialkoxysilane is vital for formation of large silsesquioxane particles. To clarify the concentration dependence of the silicification, we first monitored the light scattering intensity of **1** dissolved in pH 8 ammonium buffer solution at different concentrations (1, 10, and 40 mM). The intensity increased considerably at 40 mM within 10 h. The 10 mM solution showed a slow intensity increase over 72 h, whereas there was no significant increase at the lower concentration (1 mM) (Fig. 1B). This suggests that **1** requires a critical concentration (∼5 mM) for the formation of relatively large silica particles (Fig. S10†). In contrast, trimethylpropylsilane–TPP (**2**) showed no concentration-dependent behavior with respect to light scattering (Fig. S10†). Therefore, **1** exhibited formation of silsesquioxanes *via* hydrolytic condensation, while for the control molecule, **2**, silicification did not occur. This was further confirmed by transmission electron microscopy (TEM) (Fig. 1C and D). While the images showed small silica particles of several hundred nanometers in diameter in the case of the low concentration solution (1 mM), larger silica particles ranging from hundreds of nanometers to several micrometers in diameter were found in the high concentration solution (40 mM). Interestingly, the light scattering of **1** at pH 7.4 showed little increase even at a high concentration. It is known that the hydrolytic condensation of trialkoxysilanes can be catalyzed by both basic and acidic conditions, whereas it is stable under neutral and low ionic conditions.^19^–^21^ Thus, a high enough critical concentration and an acidic/basic environment are necessary for the effective silicification of trialkoxysilane.

**Fig. 2:**
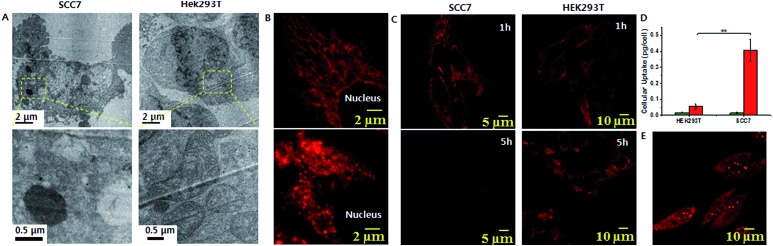
(A) Cell TEM images of **1** silica particles inside mitochondria of SCC7 cells and HEK293T cells. (B) Confocal analysis showing mitochondrial fragmentation after **1** treatment of SCC7 cells (bottom), compared with control (top). (C) Confocal microscopy images showing mitochondrial membrane depolarization using TMRM. (D) Cellular uptake of **1**. The histograms represent the mass of silicon per cell without (green) and with (red) treatment of **1**. (E) A CLSM image showing ROS generation measured by DHE in SCC7 cells (**P < 0.01, *t*-test).

The lipophilic cation (TPP) moiety can drive significantly high accumulation of trialkoxysilanes in cancerous mitochondria due to their higher negative membrane potentials than those of normal cells.^15^,^16^,^22^ This leads to the critical concentration and suitable conditions for silicification within the mitochondrial basic environment (∼pH 8).^19^,^20^ To assess intra-mitochondrial silicification, TEM experiments for SCC7 cells, a squamous cell carcinoma cell line, treated with **1** (at 10 μM for 72 h) were conducted. As shown in Fig. 2A, the cellular morphology was disrupted and normal mitochondria were not observed. Instead, large particles of ∼1 μm in diameter were observed, which might indicate the occurrence of silicification within mitochondria. In contrast, the mitochondria of HEK293T cells, a normal kidney cell line, remained unaffected by the same **1** treatment. To further investigate mitochondrial dysfunction, we observed the mitochondrial morphology through fluorescence microscopy. The elongated healthy mitochondria showed severe morphological damage with fragmentation when incubated with **1** (Fig. 2B). However, the mitochondria remained healthy when incubated with **2**. Using ImageJ software with instructor direction,^27^ we could identify mitochondrial fragmentation *via* edge detection and the average area/perimeter ratio of mitochondria, which is an index of interconnectivity. The index of interconnectivity was calculated as 0.2182 and 0.00637 in SCC7 cells treated with PBS and molecule **1**, respectively, indicating significant mitochondrial fragmentation (Fig. S19†). Depolarization of the mitochondrial membrane was observed using tetramethylrhodamine methyl ester (TMRM), which shows red fluorescence that vanishes on membrane depolarization.^23^ Red fluorescence was observed in both TMRM-labeled SCC7 cells and HEK293T cells treated with **1** after 1 h of incubation. The fluorescence faded in SCC7 cells within 5 h, whereas HEK293T cells still showed bright red fluorescence after 5 h of incubation (Fig. 2C). The confocal images revealed that **1** can selectively depolarize the mitochondrial membrane. In contrast, the fluorescence in cancer cells treated with **2** was detected even after 5 h (Fig. S12†).

We speculated that concentration-dependent biomineralization would give rise to selective mitochondrial dysfunction in cancer cells. The accumulation of **1** inside the mitochondria of cancer cells induces silicification, leading to mitochondrial dysfunction, while the concentration in the mitochondria of normal cells might not be sufficient for silicification occurrence. To investigate this, we measured the amount of Si per cell using inductively coupled plasma-optical emission spectrometry (ICP-OES). When both cell types were incubated with 25 μM **1** for 48 h, ICP-OES analysis showed that 0.408 pg per cell and 0.056 pg per cell of silica accumulated in the SCC7 cell and the HEK293T cell, respectively (Fig. 2D). This indicated that the concentration of **1** in the mitochondria of cancer cells was 7-fold greater than that in the mitochondria of normal cells (Fig. S11†). This was mainly due to the difference of mitochondrial membrane potential between normal and cancer cells.^16^ Similarly to the concentration-dependent formation of silica particles in the bulk solution (Fig. 1C and D), significantly larger silica particles were formed in the mitochondria of cancer cells due to the high accumulation of **1**. In contrast, the low concentration of **1** in the mitochondria of normal cells might not be sufficient to form large silica particles. This result was consistent with the TEM measurements (Fig. 2A). Additionally, the cellular uptake of **2** was found to be about 2-fold less efficient than that of **1** (Fig. S11†), suggesting that the formation of silica particles accelerates the accumulation of **1** unlike monomeric TPP **2**.^17^ As a consequence, these results revealed that silicification occurring at a high concentration has an important damaging effect on the mitochondria of cancer cells. Depolarization of the mitochondrial membrane leads to oxidative stress, which generates mitochondrial reactive oxygen species (ROS). Excessive production of ROS causes damage in mitochondrial DNA, membranes and proteins. Confocal analysis using dihydroethidium (DHE), which emits bright red fluorescence in response to elevated levels of ROS, showed strong red fluorescence in SCC7 cells after 5 h of incubation with **1** (Fig. 2E). In contrast, the red fluorescence was not observed in HEK293T cells (Fig S14†). Furthermore, staining with MitoSOX Red, which also emits red fluorescence in the presence of ROS, showed bright red fluorescence (Fig. S13†) indicative of mitochondrial ROS generation. However, when incubating DHE labeled SCC7 cells with **2**, red fluorescence was not observed after 5 h of incubation (Fig. S15†), implying that biomineralization induces mitochondrial dysfunction rather than the molecule itself. To confirm mitochondrial dysfunction, we analyzed the cellular ATP level as mitochondrial dysfunction would affect ATP production.^16^ The ATP activity in SCC7 cells treated with **1** decreased to 20% after 72 h of incubation (Fig S20†).

**Fig. 3:**
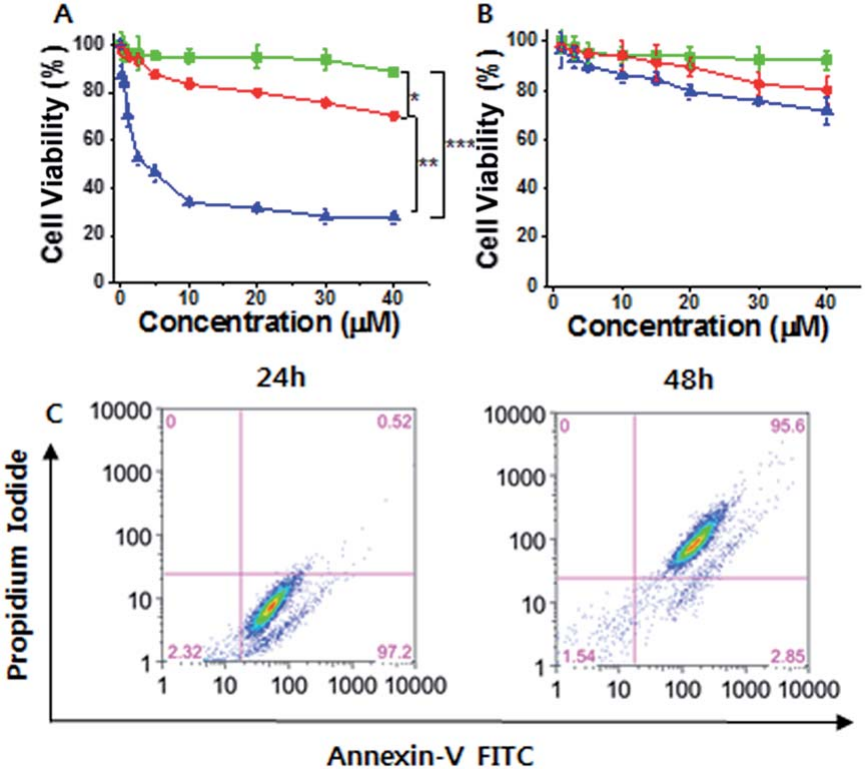
(A and B) Cell viability analysis of (A) SCC7 cells and (B) HEK293T cells treated with **1** for 24 h (green lines), 48 h (red lines) and 72 h (blue lines). (C) Flow cytogram image representing the apoptosis assay based on annexin V-FITC and PI staining of SCC7 cells treated with 25 μM **1** (*P < 0.05, **P < 0.01, ***P < 0.005, *t*-test).

Considering the cancer selectivity of mitochondrial dysfunction by **1**, we expect that this will be a cancer specific therapy since mitochondrial dysfunction induces cellular death. We analyzed whether specific silicification could affect cell viability. After 24 h of incubation, **1** exhibited low toxicity even at high concentrations (up to 40 μM). However, significant cellular toxicity was observed with a half maximal inhibitory concentration (IC_50_) of 3–4 μM after 72 h of incubation (Fig. 3A). It may be considered that this time-dependent toxicity against SCC7 cells is due to the requirement for a period over which silicification can occur. In contrast, **1** showed low toxicity towards the normal cells even after 72 h, suggesting that **1** could be used in an anticancer therapeutic approach (Fig. 3B). The cell viability results were further confirmed by propidium iodide (PI) and FITC-annexin V staining analysis.^24^ These showed the presence of annexin V but not PI in SCC7 cells treated with 25 μM **1** for 24 h, (Fig. S16†), indicative of an early apoptotic phase. Fluorescence-activated cell sorting (FACS) was used to quantitatively analyze fluorescence of FITC-annexin V/PI staining. The FACS results showed that the cells lapsed into late apoptotic stages within 48 h (Fig. 3C). As a consequence, **1** produced specific silicification inside the mitochondria of cancer cells, inducing mitochondrial dysfunction that resulted in apoptosis. In contrast, **2** had lower toxicity towards both cancer and normal cells (Fig. S22†). As another control, a TPP molecule without the silane part ((3-bromopropyl)triphenylphosphonium) also showed low toxicity towards cancer cells even after 72 h of incubation, indicating that the silicification is essential to induce apoptosis of cancer cells (Fig. S23†).

To investigate this system further, our molecular design was extended to triethoxysilane-Mor (**3**) targeting lysosomes (Scheme S2†).^25^ We wondered whether silicification in the lysosome would also be efficient, because the lysosome is much smaller so the local concentration of **3** could be significantly increased and low pH (pH 4–5) could rapidly catalyze the silicification. As a lysosome is a crucial organelle containing numerous hydrolytic enzymes, dysfunction of the lysosome could lead to cellular toxicity. However, lysosome targeting molecule **3** showed low toxicity towards cancer cells (Fig. S24†). We considered that the insignificant lysosomal stress caused by exocytosis that could induce the escape of silica particles might result in the reduced extent of lysosomal dysfunction.^26^

Mitochondria are important organelles that are associated with cellular function, ATP production, and cell death mechanisms. It is considered that mitochondria dysfunction in cells can induce apoptosis. Dysfunction of mitochondria leads to the release of cytochrome c, which is a biomarker of apoptosis. Thus, incorporating mitochondria targeting strategies into the biomineralization system would be beneficial due to the targeted delivery capability for desired therapeutics, reducing side effects. To investigate whether mitochondria targeting biomineralization shows an anticancer effect against various cancer cell lines (SCC7, MDA-MB-468, HeLa, KB and PC3 MDAMB-231), we analyzed the cell viability of these cancer cell lines. We expected that intra-mitochondrial biomineralization could also be used with other cancer cell lines. The toxicity of **1** towards a variety of cancer cell lines, such as MDA-MB-468 cells and HeLa cells, was analyzed and they showed an IC_50_ of 10–20 μM (Fig. S17, S18 and S21A–F†). Furthermore, we considered that the targeting mitochondria strategy could be a promising approach to overcome drug resistance caused by efflux proteins in cellular membranes. **1** had high toxicity against the drug resistant cell lines SNU-620-ADR cells and MCF7/ADR cells, with an IC_50_ of about 21 μM (Fig. S21G and H†). These results suggested that mitochondrial dysfunction by biomineralization could be a good method to overcome multidrug resistance. In contrast, a low toxicity was observed in the normal cells NIH-3T3. This was a result of the lower accumulation of **1** (Fig. S21I†). Overall, these results indicated that selective intra-mitochondrial silicification has an anti-cancer effect (Table 1).

**Table 1:** IC_50_ of **1** against each cell line

Tissue source	Cell line	IC_50_ (μM) for **1**
Mouse squamous cell carcinoma	SCC7	3
Human breast cancer	MDA-MB-468	12
Human cervix cancer	HeLa	15
Nasopharyngeal adenocarcinoma	KB	5
Human prostate cancer	PC3	10
Human breast cancer	MDA-MB-231	36
Human breast cancer	SKBR3	19
Dox resistance gastric cancer	SNU-620-ADR	23
Multidrug resistant breast cancer	MCF7/ADR	21
Noncancerous fibroblast	HEK293T	71
Noncancerous fibroblast	NIH-3T3	71

**Fig. 4:**
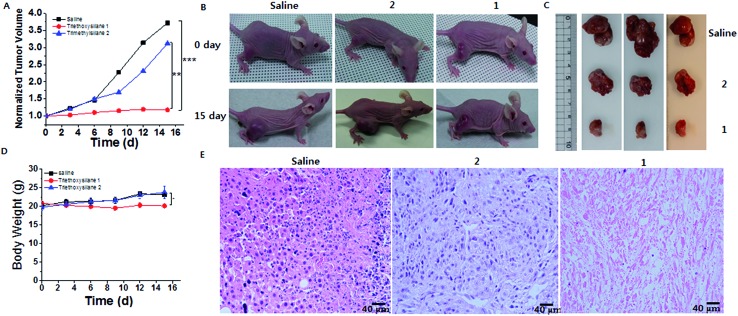
Anticancer therapy by intra-mitochondrial biomineralization. (A) Normalized tumor growth curves in the control, **1**, and **2** (control). (B and C) Images of mice and tumors before and after the different treatments. (D) Body weights of mice after the different treatments. (E) H&E staining image of a tumor section (*P < 0.05, **P < 0.01, ***P < 0.005, *t*-test).

Finally, to validate the silicification at tissue level, *in vivo* evaluations were performed. Balb/C nude mice (purchased from Orientbio, Korea) weighing ∼20 g (7 weeks old, female) were kept in cages and fed periodically following the UNIST-IACUC guidelines for the care and use of laboratory animals. All animal experiments were conducted under protocols approved by the Institutional Animal Care and Use Committee of the Ulsan National Institute of Science and Technology (UNIST). After the establishment of tumors, mice were treated with saline, **2**, and **1** by peritumoral injection once every 3 days for 16 days (day 0, 3, 6, 9 and 12). A total of 12 mice were allocated to three groups treated with **1** (50 mg kg^–1^ in PBS), **2** (50 mg kg^–1^ in PBS) and saline as control. ICP-OES analysis showed that 1.75 mg g^–1^ tumor, 5.75 mg g^–1^ tumor, and 10.3 mg g^–1^ tumor of silica accumulated in the saline, **2** and **1** groups, respectively (Fig. S25†). These *in vivo* tissue results were consistent with the cell-level *in vitro* results. In addition, *ex vivo* data showed that silicon accumulated well inside the tumors, compared with normal tissue (Fig. S25†). Given the targeting silicification, we evaluated the *in vivo* therapeutic efficiency of the silicification in mice bearing SCC7 tumors. As shown in Fig. 4A–C, treatment with **2** showed comparable tumor growth inhibition with that in the control. In contrast, tumor growth was suppressed by a formulation of **1**, and H&E staining showed tumor remission after triethoxysilane **1** treatment, suggesting that the silicification has a significant anticancer effect (Fig. 4E). Additionally, the normal organs remained healthy after triethoxysilane treatment (Fig S26†). Meanwhile, a noticeable change of body weight and even of body weight with tumor weight subtracted was not observed in either group, indicating that the silicification was biologically friendly (Fig. 4D and S27†). Furthermore, to investigate the fate of **1***in vivo*, we analyzed the amount of silicon in urine samples, which were collected into separate tubes at 4 h, 12 h, 24 h and 72 h after administration of **1** (50 mg kg^–1^). ICP-OES analysis showed that 7.6 μg mL^–1^ of silicon was excreted into urine at 4 h. However, an insignificant amount of silicon was observed in urine collected after 12 h, suggesting that **1** would be excreted into urine within 12 h (Fig S28†).

## Conclusions

In summary, we developed a mitochondria-targeting silicification system using **1** that selectively eliminates cancer cells. **1** produced concentration dependent silicification under acidic/basic conditions, revealing that effective silicification occurs only at high concentrations. The lipophilic cation (TPP) moiety of this system drives the accumulation of **1** inside the mitochondria, providing the effective conditions for silicification. This specific silicification induces the depolarization of the mitochondrial membrane, disrupting it and generating ROS. As mitochondria are the power sources of cells, the dysfunction of mitochondria leads to apoptosis in cancer cells. At the *in vivo* level, this system showed inhibition of tumor growth. Thus, our novel approach through organelle-specific biomineralization provides a new strategy for the treatment of cancer cells.

## Conflicts of interest

There are no conflicts to declare.

## Supplementary Material

Supplementary information
