# Supplementary material for: Intra-mitochondrial biomineralization for inducing apoptosis of cancer cells
Source: Chem Sci. 2018 Jan 25;9(9):2474–9. doi: 10.1039/c7sc05189a (PMC5909330; doi:10.1039/c7sc05189a)
Supplement: Supplementary file 1 [file SC-009-C7SC05189A-s001.pdf]

Electronic Supplementary Material (ESI) for Chemical Science.
This journal is © The Royal Society of Chemistry 2018

Supporting Information

Intra-Mitochondrial Biomineralization for inducing apoptosis of cancer cells.

Sangpil Kim, L‡. Palanikumar‡, Huyeon Choi, M. T. Jeena, Chaekyu kim*, Ja-Hyoung Ryu*

Experimental Procedures

S1. Synthesis and characterization

S1.1. Materials and characterization

(3-chloropropyl)trichlorosilane was purchased from Alfa Aesar(British), (4-carboxybutyl)triphenyl phosphonium bromide was obtained from Sigma Aldrich (USA). 3-aminopropyltriethoxysilane was obtained from TCI (Japan). (3-chloropropyl)trimethylsilane was purchased from Sigma Aldrich (USA). 2-Propanol, Pentane and 1-Butanol, Chloroform HPLC grade obtained from from Sigma Aldrich (USA). Sodium azide was purchased from TCI (Japan). Palladium 10% on carbon was obtained from TCI (Japan). N,N-dimethylformamide (DMF), Dichloromethane (DCM), Methanol HPLC grade was purchased form DAEJUNG (South Korea). N,N'-Dicyclohexylcarbodiimide (DCC), 4-Dimethylaminopyridine (DMAP) were obtained from Gl Biochem (Shanghai) Ltd (China). Molecular sieves 4A, 4-8mesh was purchased from SAMCHUN CHEMICALS (South Korea). Methanol, Dichloromethane, Chloroform, Diethyl ether were obtained from SK Chemical (South Korea). Trialkoxysilane derivatives were characterized using 400MHz FT-NMR (Agilent Technologies and Bruker). Silica nanoparticles were characterized by TEM (JEM-1400), Cold FE-SEM (Hitachi High-Technologies), Zetasizer (Malvern ZS).

S1.2.1 Trialkoxypropylsilane-TPP synthesis

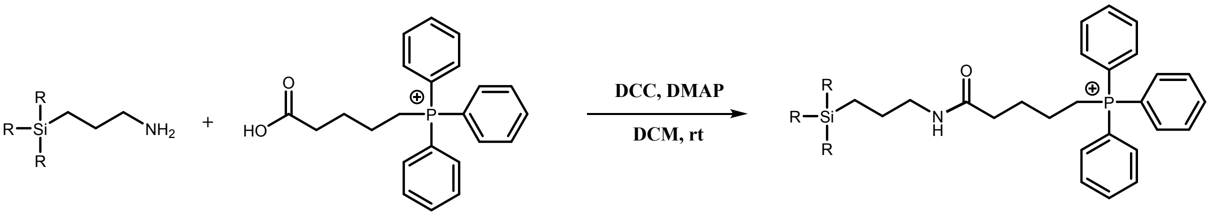

1, R = OCH_2CH_3 **2**, R = CH_3

Scheme S1. Schematic representation for synthesis of trialkoxysilane-TPP.

Triethoxyproprylsilane-TPP (1) : (4-carboxybutyl)triphenylphosphonium bromide (1.00g, 2.26mmol), dicyclohexylcarbodiimide (0.560g, 2.71mmol) and 4-Dimethylaminopyridine (0.055g, 0.450mmol) were dissolved in dry DCM (20mL). Then, 3-aminopropyltriethoxysilane (0.5g, 2.26mmol) was added carefully in mixture solution. After stirring at room temperature for 5h, the mixture was filtered and the residue was purified by size exclusion chromatography. Product **1** was a yellow oil. Yield : 0.703g (55%). ^{1}H NMR (400 MHz, $CDCl_3$): δ0.6 (t,2H), 1.22(t,9H), 1.26(m,2H), 1.56-1.71(m,4H), 1.94(m,2H), 2.71(t,2H), 3.17(q,2H), 3.81(q,6H), 7.67-7.81(m,15H), 8.22(t,1H) ^{13}C NMR (400MHz, $CDCl_3$): δ 7.94, 18.34, 22.91, 26.06, 34.11, 42.24, 58.34, 117.75, 118.60, 130.41, 133.62, 135.05, 172.72. ESI-MS: 567.12[M]

Trimethylproprylsilane-TPP (2) : (4-carboxybutyl)triphenylphosphonium bromide (1.01g, 2.28mmol), dicyclohexylcarbodiimide (0.565g, 2.74mmol) and 4-Dimethylaminopyridine (0.055g, 0.450mmol) were dissolved in dry DCM (20mL). Then, 3-aminopropyltrimethylsilane (0.3g, 2.28mmol) was added carefully in mixture solution. After stirring at room temperature for overnight, the mixture was filtered and the residue was purified by size exclusion chromatography.[1] Product **2** was a yellow oil. Yield: 0.739g (68%). ^{1}H NMR (400 MHz, $CDCl_3$): δ0.01(s,9H) 0.45 (t,2H), 1.25(m,2H), 1.56-1.71(m,4H), 1.94(m,2H), 2.56(t,2H), 3.65(q,2H), 7.67-7.81(m,15H), 8.22(t,1H) ^{13}C NMR (400MHz, $CDCl_3$): δ 0.13, 15.86, 24.72, 27.67, 35.01, 53.20, 119.21, 120.18, 132.31, 135.34, 136.83, 174.64. MALDI-TOF: 476.29[M]

S1.2.2 Trialkoxypropylsilane-Morpholine synthesis

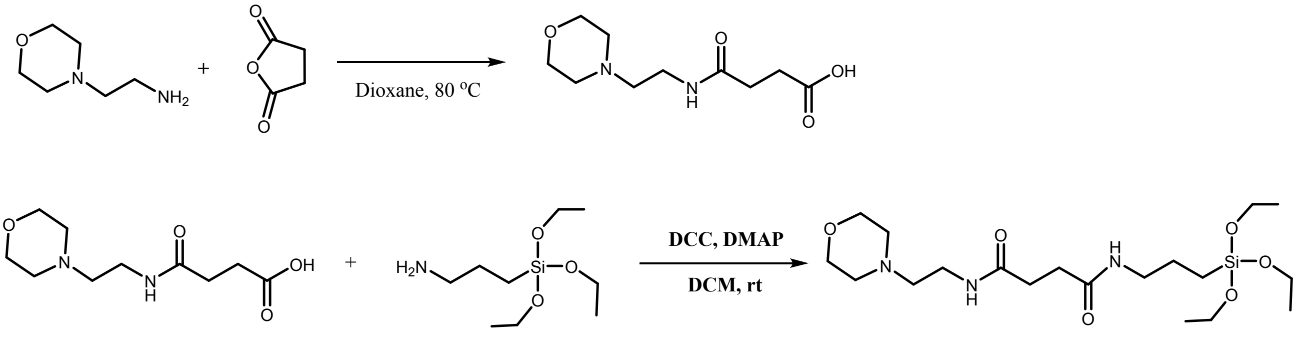

3

Scheme S2. Schematic representation for synthesis of trialkoxysilane-Mor

Triethoxysilane-Mor (3): Succinic anhydride (0.230 g, 2.3 mmol) was dissolved in 2mL of dioxane and 2-aminomorpholine (0.300 g, 2.3 mmol) was slowly added. After that, solution was warmed at 80 ℃ for 30 min. The white crystals were filtered off, dried, and recrystallized from dioxane. The resulting product was a white powder. The resulting product(0.1 g, 0.434 mmol), dicyclohexylcarbodiimide (0.107 g, 0.521 mmol) and 4-Dimethylaminopyridine (0.010g, 0.087 mmol) were dissolved in dry DCM (10mL). Then, 3-aminopropyltrimethylsilane (0.096 g, 0.434 mmol) was added carefully in mixture solution. After stirring at room temperature for overnight, the mixture was filtered and the residue was purified by size exclusion chromatography.[1] Product **3** was a yellow solid. Yield: 0.122 g (65%). ^{1}H NMR (400 MHz, $CDCl_3$): δ0.63 (t,2H), 1.22(t,9H), 1.77(m,2H), 1.45(m,6H), 2.56(t,2H), 2.52(m,4H), 3.22(q,2H), 3.33(q,2H), 3.71(t,4H), 3.82(q,6H) ^{13}C NMR (400MHz, $CDCl_3$): δ 7.74, 18.24, 25.29, 31.78, 35.78, 41.95, 53.30, 56.97, 58.38, 66.72, 77.36. ESI-MS: 434.6[M]

S1.3. Silica particles characterization by TEM.

A drop of each sample (10 µM) in aqueous solution was placed on a 300-mesh formvar/carbon-coated copper grid and allowed to evaporate under ambient conditions. The specimen was observed with a JEM-1400 operating at 120 kV. The data were analyzed with Gatan Digital Micrograph program. For preparation of cellular TEM samples, HEK293T cells and SCC7 cells were seeded and incubated on 15-mm diameter Theramanox coverslips (Nalge Nunc International), placed in 24-well plates (50,000 cells in 1mL serum containing media) for 24 h before experiment. The media was replaced by 10 µM triethoxysilane **1** in serum containing media and incubated for 72 h. The medium containing triethoxysilane **1** was discarded and the cells were completely washed with 0.1 M sodium phosphate buffer (pH 7.0) three times. The cells were then fixed in 2% glutaldehyde with 5% sucrose in 0.05 M sodium phosphate buffer (pH 7.0) for 30 min and washed with 0.05M sodium phosphate buffer (pH 7.0) containing 5% sucrose, three times over 30 min. The cells were postfixed in 1% osmium tetraoxide with 5% sucrose in 0.05 M sodium phosphate buffer solution (pH 7.0) for 1 h and then rinsed with third distilled water three times. They were dehydrated in a graded series of acetone (20% steps) and embedded in epoxy resin. The resin was polymerized at 80 ℃ for 12 h. Ultrathin sections (70 nm) were obtained with CR-X ultramicrotome then imaged with JEM-1400 operating at 120 kV. When sample was stained, a drop of uranyl acetate solution (2 wt%) placed onto the surface of the sample-loaded grid. The sample deposited about 1 min at least, and excess solution was wicked off by filter paper.

S1.4. Light scattering intensity measurements

To determine variation of light scattering intensity as results of silicification, several concentrations (1 mM, 10 mM, 40 mM) of triethoxysilane **1** and 40 mM trimethylsilane **2** were prepared both in 0.2M ammonia buffer solution (pH 8, 7.4). The solutions were stirred at 37 ℃ for 72h. The light scattering intensity was measured by Zetasizer ZS series (United Kingdom). The intensity increased

considerably at 40 mM and 10 mM solution over 72 h, whereas those were not significant extent at 1 mM solution of 1 and 40 mM solution of 2 in pH 8 ammonium buffer solution. In contrast, significant variation was not observed in pH 7.4 solution.

S2. In vitro experiment.

S2.1. Materials and methods.

All chemicals used were purchased from Sigma Aldrich, Korea unless otherwise stated. Cell culture reagents were obtained from Life Technologies, Korea. The tetra methyl rhodamine methyl ester (TMRM) was purchased from Santa Cruz Biotechnologies, Korea. MitoSox, dihydroethidium (DHE), annexin V, propidium iodide (PI), and Alexa Fluoro 488 were purchased from Life Technologies, Korea. The confocal laser scanning microscopy images were taken with Olympus FV 1000 confocal microscopes.

S2.2. Cell culture and cell viability analysis.

Human cancer cells originating from the prostate (PC3), cervix (HeLa), breast (SK BR 3, MDA MB 231, MCF7/ADR and MDA MB 468), liver (SK HEP), dox resistance gastric cancer (SNU-620 ADR), nasopharyngeal adenocarcinoma (KB), and mouse squamous cell carcinoma (SCC 7), as well as noncancerous fibroblast (HEK293T and NIH3T3) cells, were obtained and cultured in DMEM, L-15, or RPMI (Life Technologies) containing 10% fetal bovine serum (FBS; Life Technologies) and 1% penicillin/streptomycin (Life Technologies) at 37°C in a humidified atmosphere of 5% CO_2. For cytotoxicity analysis, cells were cultured in 96-well Nunc (Thermo Fisher Scientific Inc.) plates, seeding with cells at a density of 5 x 10^3 cells/well; the cells were allowed to settle for 24 h with incubation at 37°C under 5% CO_2 in the respective growth medium (e.g., RPMI 1640, DMEM, or L-15). Different concentrations of triethoxysilane **1,** trimethylsialne **2** (0.1, 2, 3, 5, 10, 15, 20, 25, 30, 35 and 40 µM/well) were treated. Cell viability was measured at 24 h, 48 h and 72 h using the Alamar blue assay, with each data point measured in triplicate. Fluorescence measurements were obtained using a plate reader (Tecan Infinite Series, Germany) by setting the excitation wavelength at 565 nm and monitoring emission from the 96-well plates at 590 nm.

S2.3. Mitochondrial fragmentation analysis.

SCC7 cells were seeded on a Lab Tek II four well slide chamber at 60-80% confluence in RPMI and DMEM media supplemented with 10% FBS and 1% penicillin/streptomycin at 37°C and 5% CO_2. The cells were labeled with Mitotracker deep red FM (Invitrogen, Thermoscientific) incubated with 80 µM of different triethxoysilane **1** and trimethylsilane **2**, or DMSO (control) for 24h, and analyzed using an FV1000 laser confocal scanning microscope (Olympus).

S2.4. Confocal imaging of TMRM depolarization.

SCC7 cells and HEK293T cells were seeded on a Lab Tek II four well slide chamber at 60-80% confluence in RPMI and DMEM media supplemented with 10% FBS and 1% penicillin/streptomycin at 37°C and 5% CO_2. The cells were labeled with 200 nM TMRM, incubated with 40 µM of triethoxysilane **1** and trimethylsilane **2**, or DMSO (control) for different time points, and analyzed using an FV1000 laser confocal scanning microscope (Olympus).

S2.5. Confocal imaging of ROS generation in mitochondria and nucleus.

SCC7 and HEK293T cells were seeded on a Lab Tek II two wells chamber cover glass at 90% confluence in RPMI and DMEM media supplemented with 10% FBS and 1% penicillin/streptomycin and incubated at 37°C under 5% CO_2. After incubation with triethoxysilane **1,** trimethylsilane **2** at 40 µM for different time intervals, by following the manufacturer's protocol (MitoSOX, M36008); the cell culture medium was then replaced with 1 to 2 mL of 5 µM MitoSOX reagent working solution to cover the adherent cells. The cells were then incubated for 10 minutes at 37°C, protected from light. The cells were then washed gently and analyzed under a FV1000 laser confocal scanning microscope. Similarly, the dihydroethidium (DHE) assay was performed to evaluate ROS-related cytotoxicity by following the manufacturer's protocol (Santa Cruz, SC-204724A); the DHE was imaged using a FV1000 laser confocal scanning microscope.

S2.6. Cellular uptake and apoptosis analysis.

Carcinoma (SCC 7), as well as noncancerous fibroblast (HEK293T and NIH3T3) cells, were obtained and cultured in DMEM, L-15, or RPMI (Life Technologies) containing 10% fetal bovine serum (FBS; Life Technologies) and 1% penicillin/streptomycin (Life Technologies) at 37°C in a humidified atmosphere of 5% CO_2. After incubation with 25 µM triethoxysilane **1** and trimethylsilane **2** for 48h, cells were washed with PBS solution three times. After centrifugation, the cell was washed once and then, 2% (v/v) HNO_3 in aqueous solution was added to allow dissolution of the cells with ultrasound[2]. Theses clear solution was subjected to analysis by inductively coupled plasma optical emission spectroscopy (ICP-OES, Varian 700-ES).

*Intracellular amount of silicon per cell = cellular uptake (pg)／Cell number.

We speculated that the average cell volume of single SCC7 cells and HEK293T cell were caculated as 4000 μm^3. We determined the mitochondrial concentration using volume ratio of both cell to mitochondria as 0.06.

Flow cytometry analysis to check the apoptosis/necrosis-dependent cell death. SCC7 cells were seeded on a Lab Tek II two cell chambered cover glass at 90% confluence in RPMI and DMEM media supplemented with 10% FBS and 1% penicillin/streptomycin at 37°C under 5% CO_2. The cells were then incubated with 25 µM triethoxysilane **1** and trimethylsilane **2** for 24 h, 48 h and gently washed with ice cold PBS. The cell culture medium was then replaced with Alexa Fluor 488-conjugated annexin V and propidium iodide (PI) in 0.4 mL of annexin binding buffer and incubated for 15 min at RT as per the manufacturer's protocol (Life Technologies, V13241). The stained cells were then analyzed by flow cytometry (FACS caliber, BD Bioscience), with the fluorescence emission measured at 530 nm (i.e., FL1) and >575 nm (i.e., FL3).

S3. In vivo-experiment.

After a week of acclimation period, SCC7 cancer cells (~ 1 x 10^7 cells/100 µL) were subcutaneously injected to induce the tumor growth. A total of 12 healthy mice with similar tumor volume of 100 mm^3 were allocated to three groups with a) phosphate buffered saline (PBS) b) triethoxyproprylsilane-TPP (50 mg/Kg, in PBS) and c) trimethylproprylsilane-TPP (50 mg/Kg, in PBS). The samples were injected by intraperitoneal (peritumoral region) mode for every 3 days (day 0, 3, 6, 9 and 12 days). Body weights and tumor volume change were observed and recorded carefully throughout the experiment period. On day 16, tumor tissues were recovedred from the necrospsy were fixed in 10% neutral buffered formalin, embedded in paraffin, sectoned and stained with hematoxylin and eosin (H & E) for histological examination using standard techniques. After H & E staining, the slides were fixed using DPX mountant and photos were taken using olympus optical microscope.

Supplementary Figures

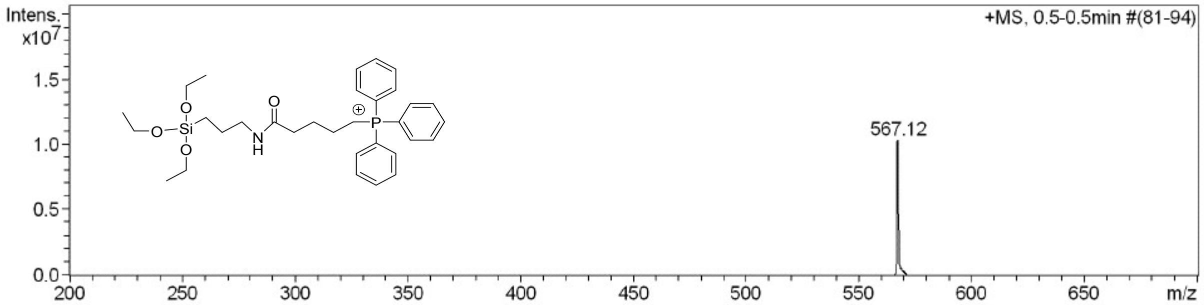

Fig. S1. Mass of triethoxysilane **1** was analyzed by ESI-MS.

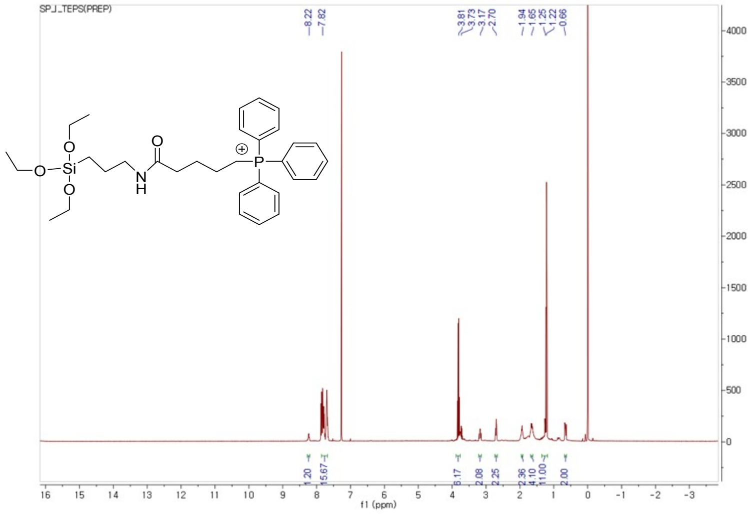

Fig. S2. ^{1}H NMR spectra of triethoxysilane **1.**

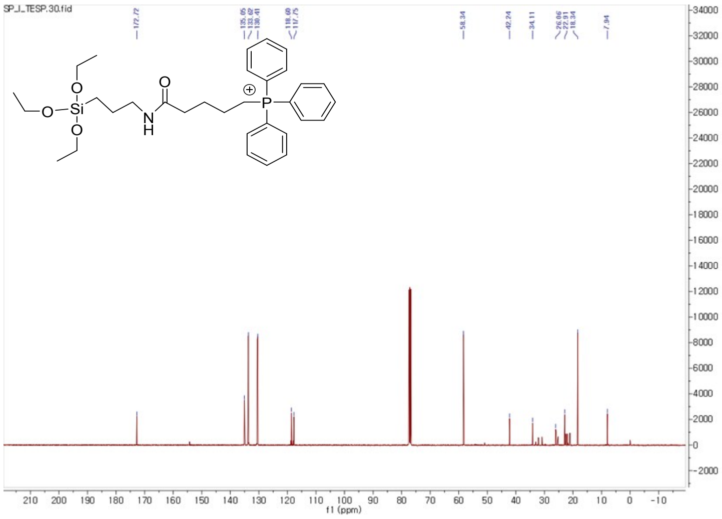

Fig. S3. ^{13}C NMR spectra of triethoxysilane **1.**

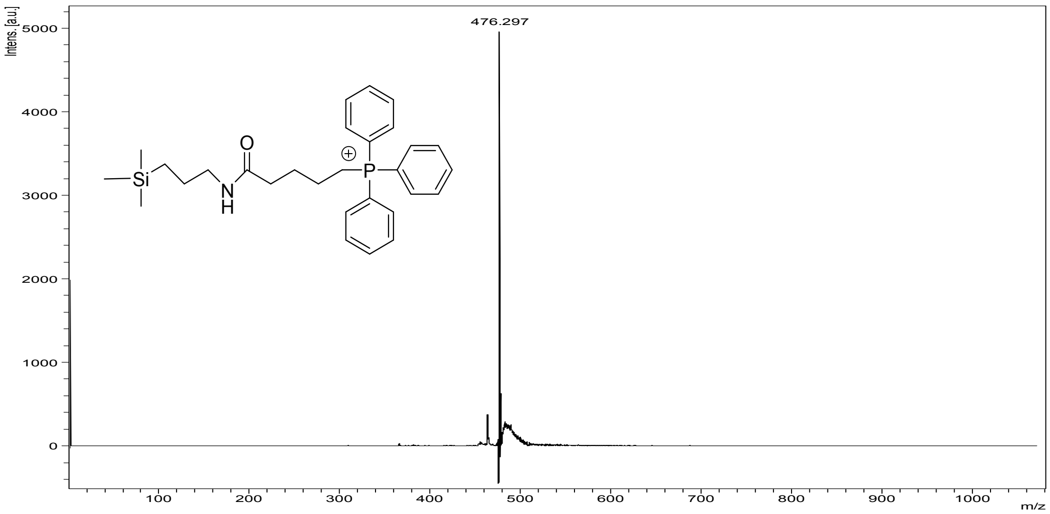

Fig. S4. Mass of trimethylsilane **2** was analyzed by MALDI-TOF.

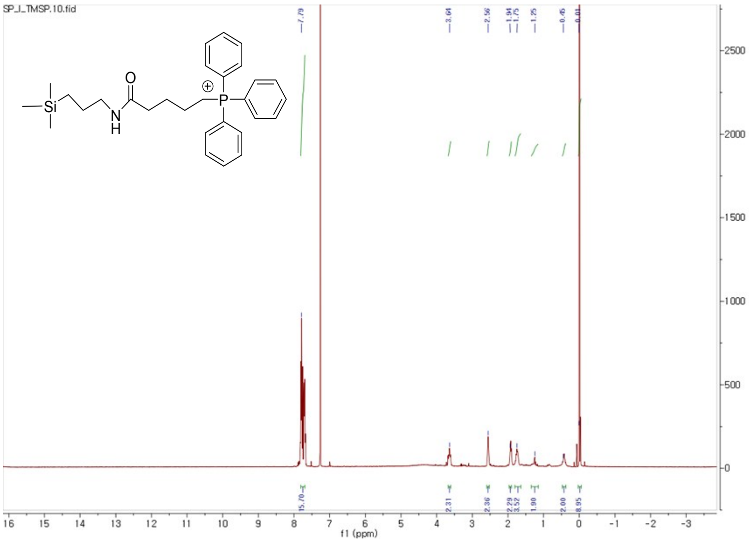

Fig. S5. ^{1}H NMR spectra of trimethylsilane **2.**

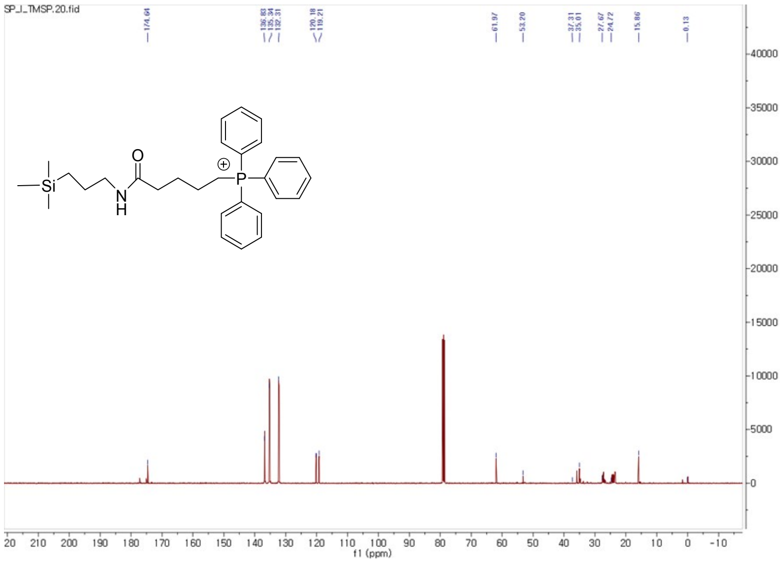

Fig. S6. ^{13}C NMR spectra of trimethylsilane **2.**

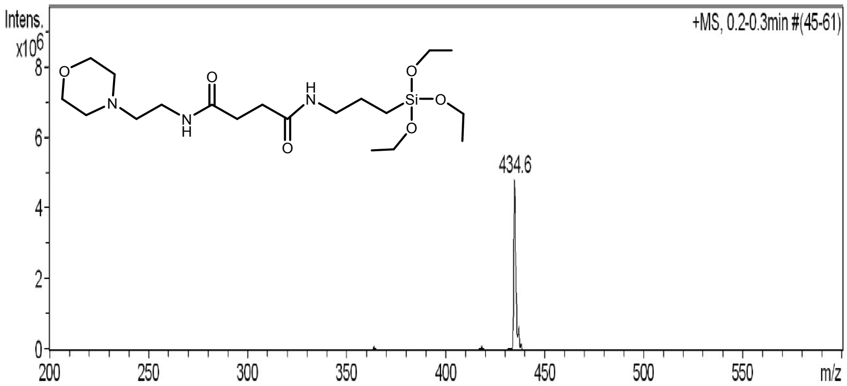

Fig. S7. Mass of triethoxysilane-Mor **3** was analyzed by ESI-MS.

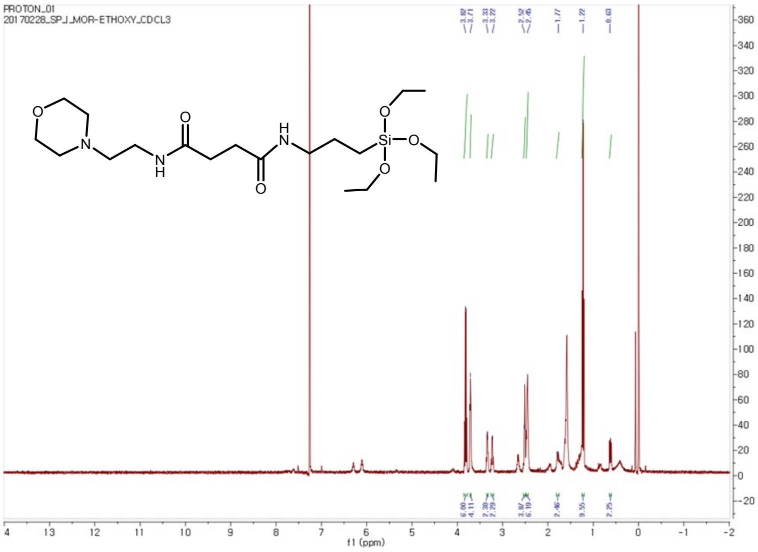

Fig. S8. ^{1}H NMR spectra of triethoxysilane-Mor **3**

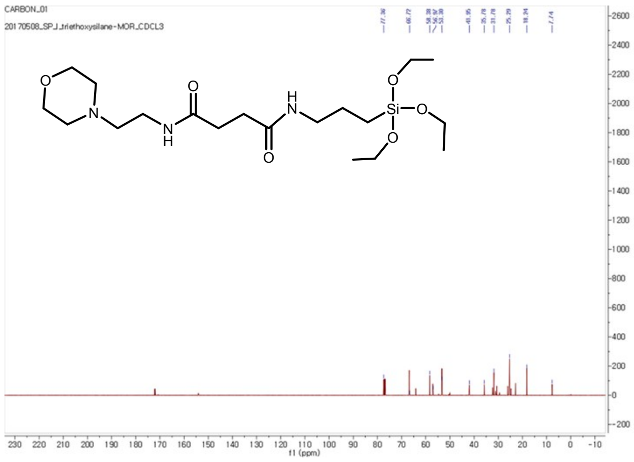

Fig. S9. ^{13}C NMR spectra of triethoxysilane-Mor **3**

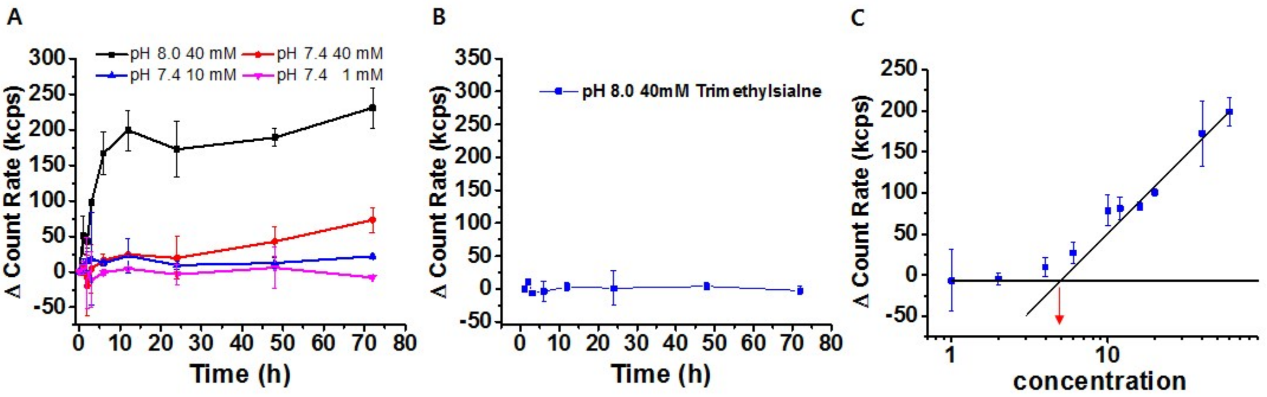

Fig. S10. A) Time-dependent light scattering intensity of **1** (1, 10, 40 mM) in ammonium buffer for 72h B) Light scattering intensity analysis of 40 mM trimethylsilane **2** dissolved in pH 8 ammonium buffer solution. C) The variation of light scattering intensity with different concentration of triethoxysilane **1** at 24h, representing critical concentration to occur effective silicification in ammonium buffer solution.

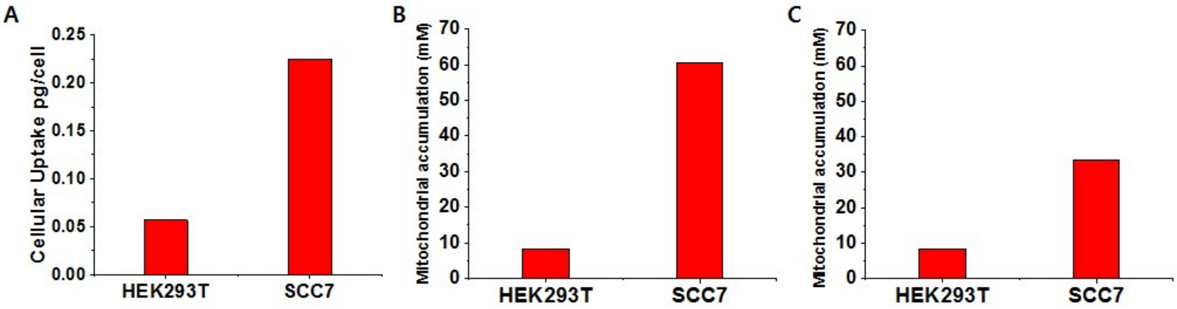

Fig. S11. A) Amount of silicon per cell in SCC7 cells and HEK293T cells after 25 μM trimethylsilane**2** treatment for 48h. (B, C) Silicon concentration of B) triethoxysilane **1** and C) trimethylsilane **2** inside mitochondria of HEK293T cells and SCC7 cells.

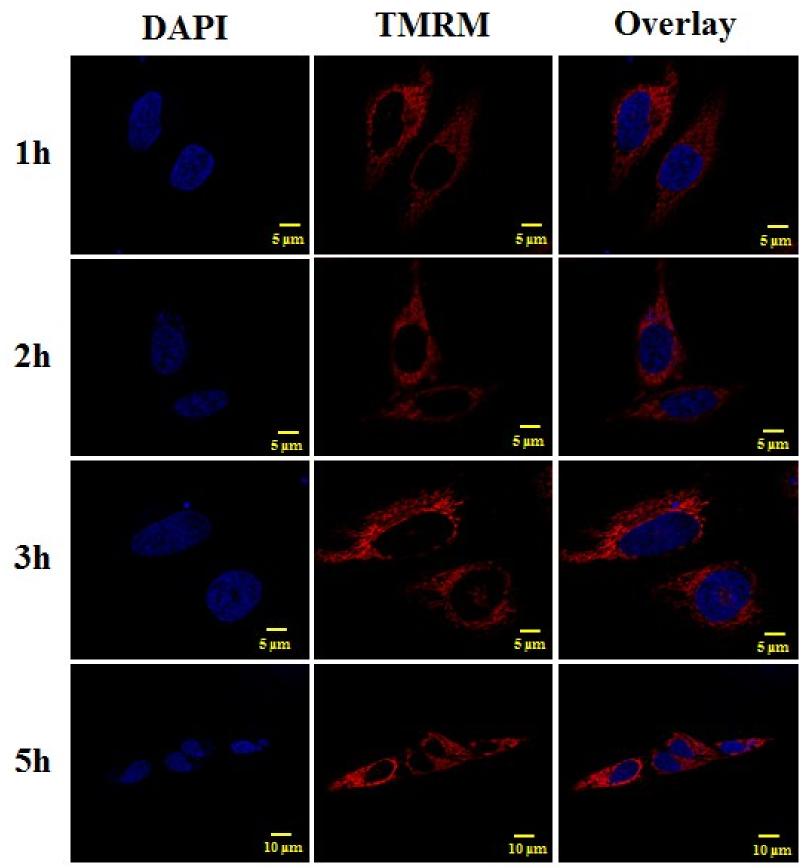

Fig. S12. CLSM image showing mitochondrial membrane depolarization analysis by using TMRM in SCC7 cells with 40 µM triethoxysilane **2** treatment for 5h.

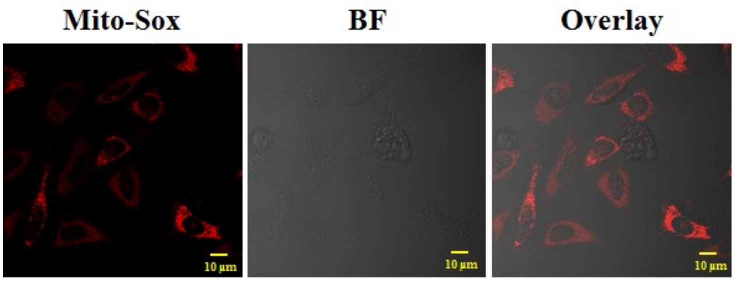

Fig. S13. Confocal image to check the mitochondria-ROS production using Mito-Sox in SCC7 cells treated with 40 µM triethoxysilane **1** for 5h.

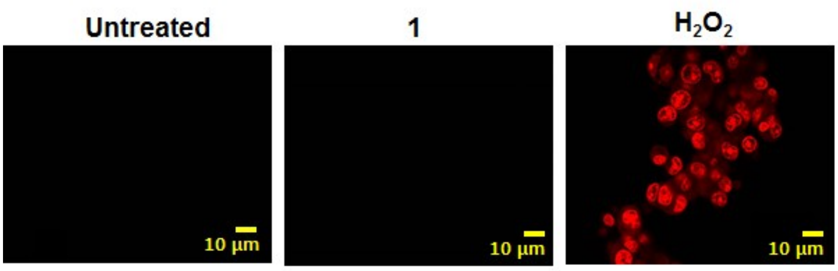

Fig. S14. CLSM image representing ROS generation analysis for HEK293T cells after 40 µM 1 treatment for 5h.

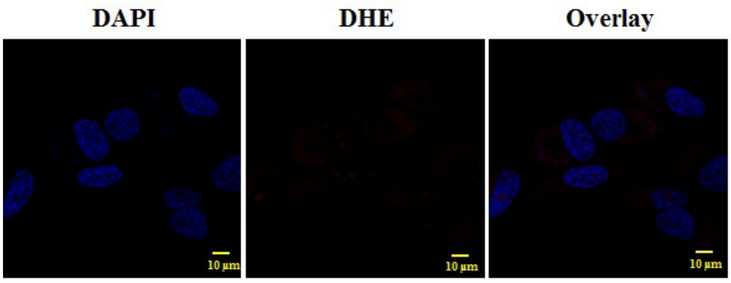

Fig. S15. CLSM image representing ROS generation analysis for SCC7 cells after 40 µM trimethylsilane **2** treatment for 5h.

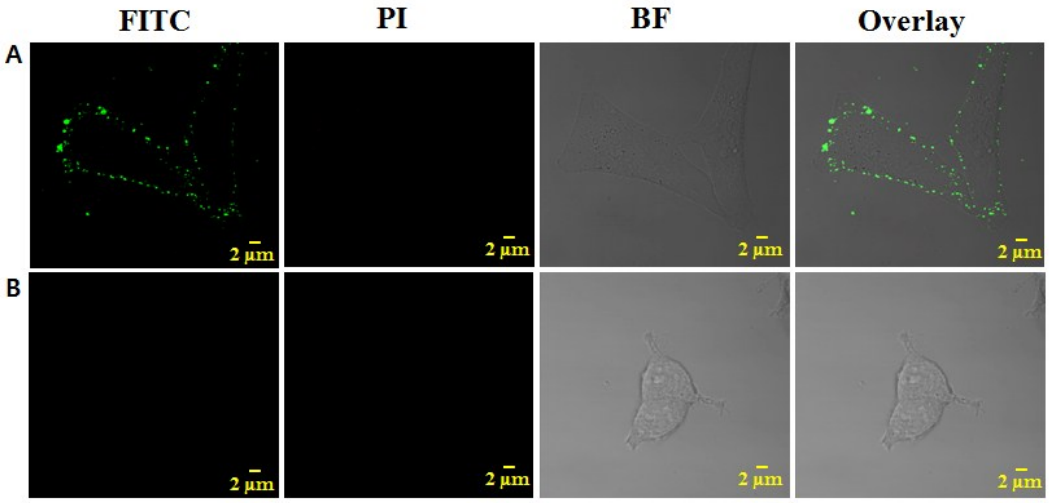

Fig. S16. Apoptosis was measured by Confocal image in A) SCC7 Cells and B) HEK293T cells after 24h incubation for 40 µM concentration triethoxysilane **1** stained with Annexin-V-FITC/propidium iodide.

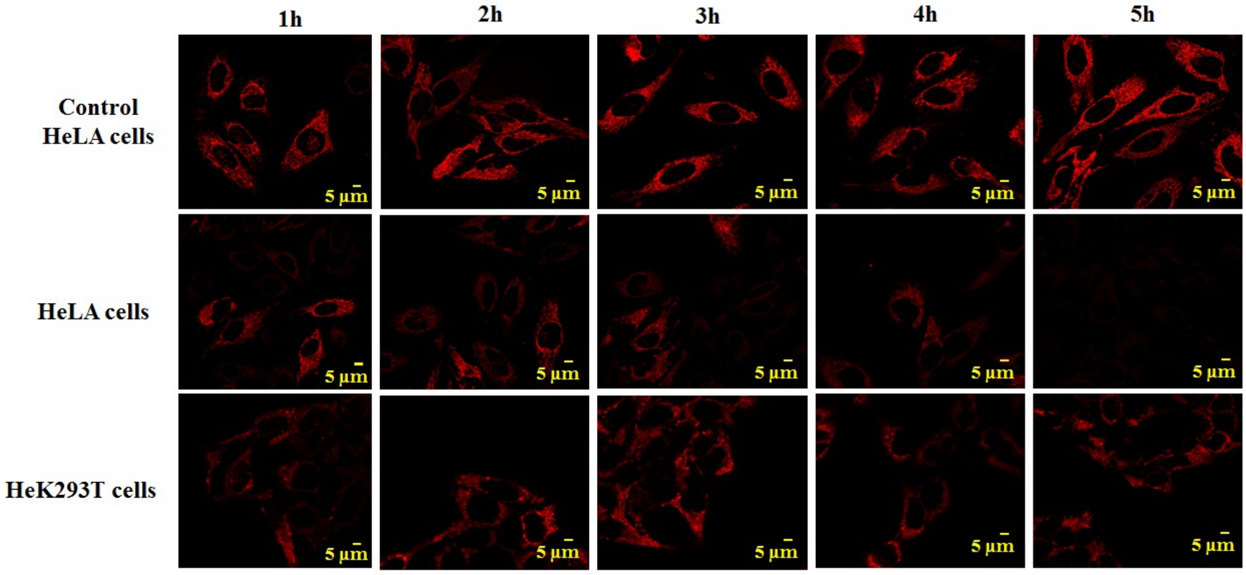

Fig. S17. CLSM image representing TMRM depolarization analysis in HeLa cells with and without 75 µM triethoxysilane **1** treatment and HEK293T cells for 5h.

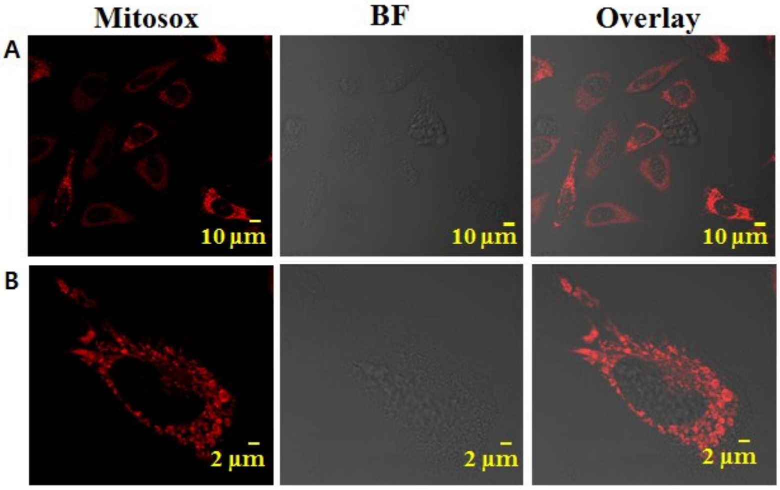

Fig. S18. A) Confocal imaging to check the mitochondria-ROS production and B) Magnification image of mitochondria staining Mito-sox in HeLa cells with 25 µM triethoxysilane **1** treatment for 38h.

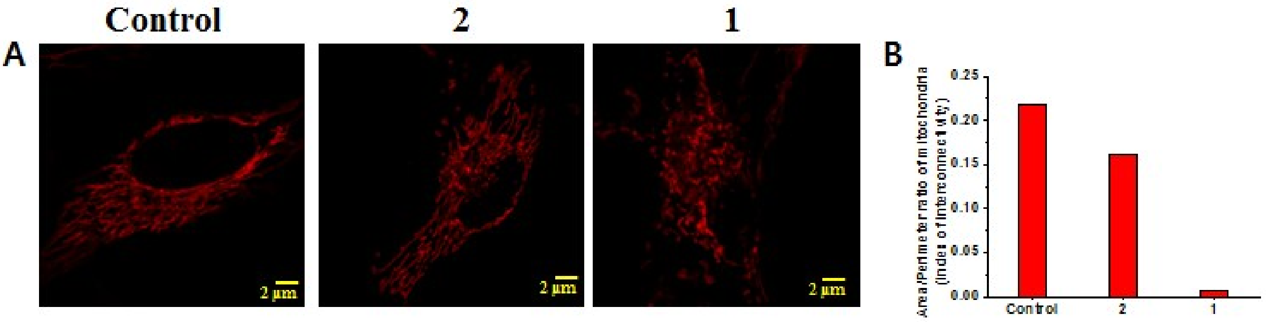

Fig. S19. A) CLSM image representing mitochondria fragmentation of SCC7 cells with 40 µM triethoxysilane **1** treatment for 12h. B) Quantification of mitochondria fragmentation.

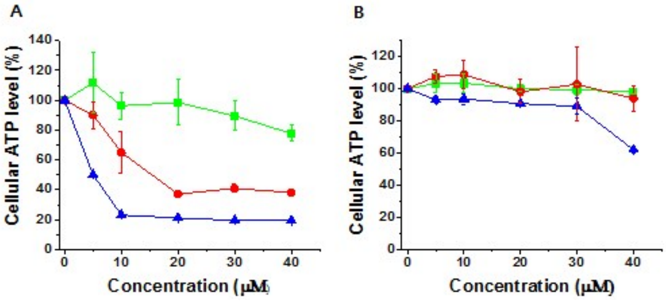

Fig. S20. Concentration-dependent ATP level curve of triethoxysilane 1 in A) SCC7 cells and B) HEK293T cells for 24h (green lines), 48h (red lines), 72h (blue lines).

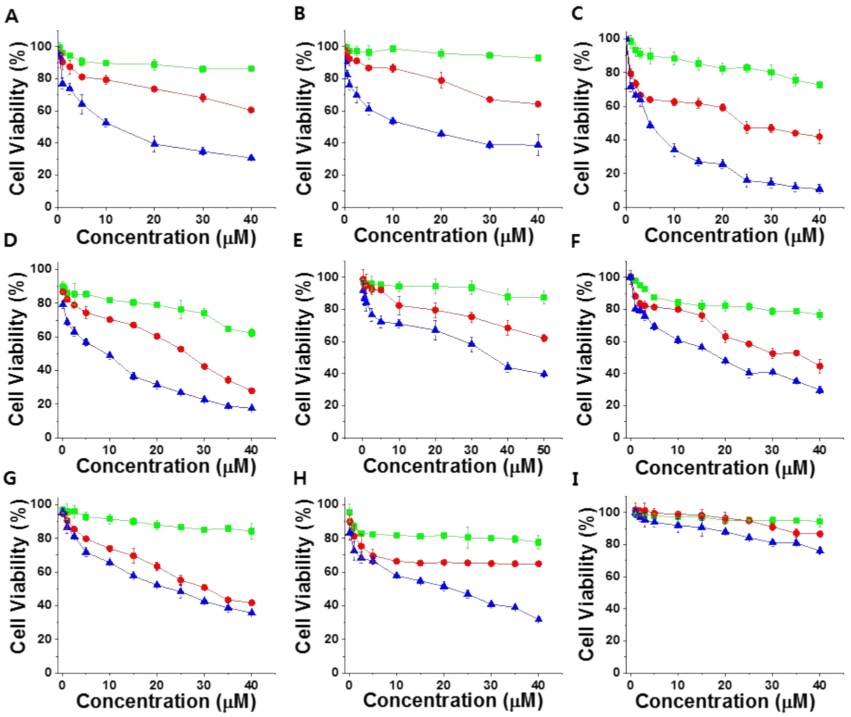

Fig. S21. Cell viability analysis of A) MDA-MB-468 cells and B) HeLa cells, C) KB cells, D) PC3 cells, E) MDA-MB-231 cells, F) SKBR3 cells, G) SNU-620-ADR cells H) MCF7/ADR cells, I) NIH-3T3 cells after incubation with triethoxysilane **1** for 24h (green lines), 48h (red lines), 72h (blue lines).

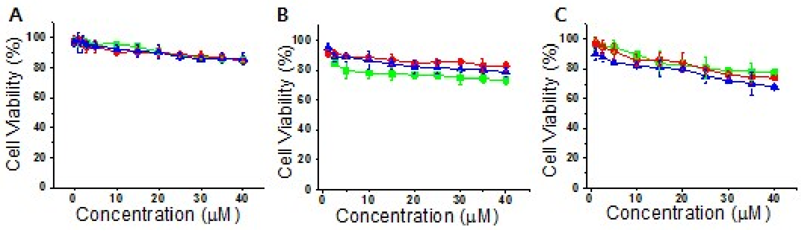

Fig. S22. Cell viability analysis of A) SCC7 cells, B) HeLa cells and C) HEK293T cells after incubation with different concentrations of trimethylsilane **2** for 24h (green lines), 48h (red lines), 72h (blue lines).

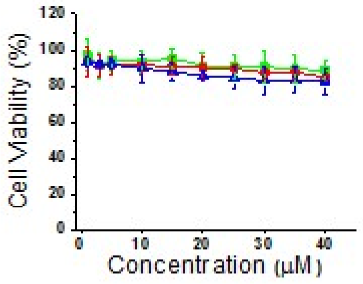

Fig. S23. Cell viability analysis of SCC7 cells after incubation with different concentration of (3-bromopropyl)triphenylphosphonium for 24h (green lines), 48h (red lines), 72h (blue lines).

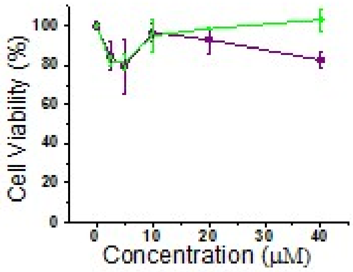

Fig. S24. A) Cell viability analysis of HeLa cells after incubation with different concentration of triethoxy-Mor **3** for 24h (green lines), 96h (purple lines).

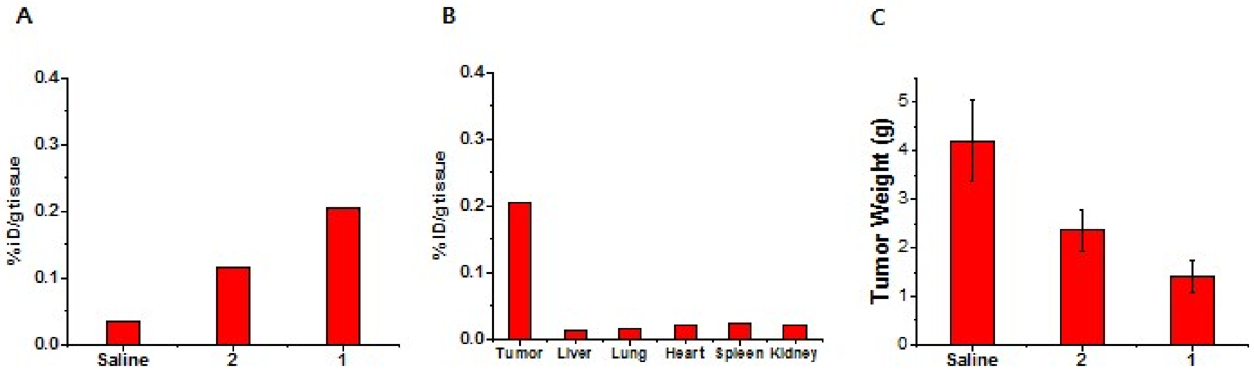

Fig. S25. A) The histogram representing the %ID/g tumor. B) The histogram representing the %injection dose in tissue. C) Weight of tumor after the different treatments for 15 days.

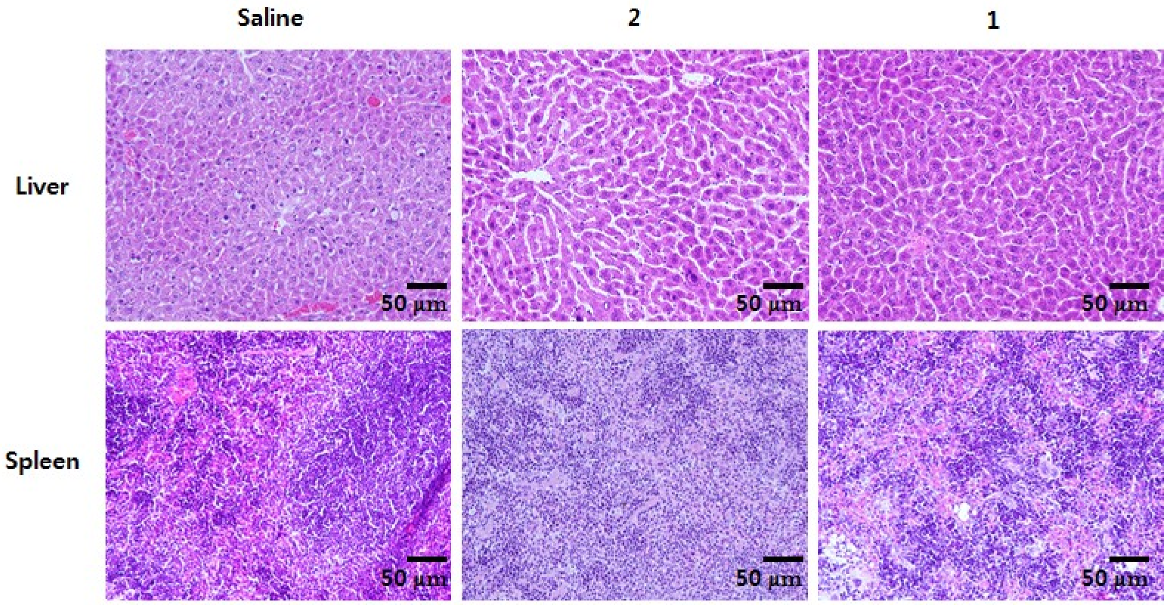

Fig. S26. H&E staining image of organs (liver, spleen) section treated with saline, **2**, and **1**

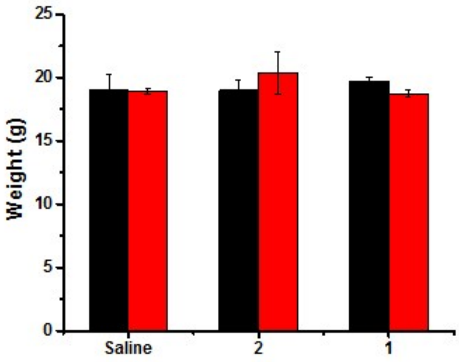

Fig. S27. The histogram representing the body weight that subtracted with tumor weight at 0 day (black bar) and 15 day (red bar).

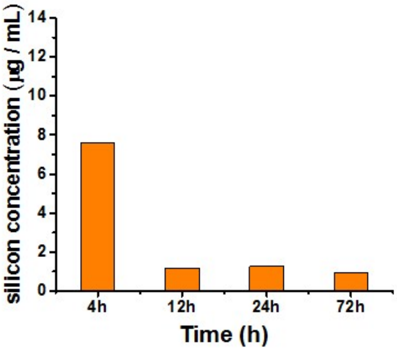

Fig. S28. The histogram representing the mass of silica/mL urine.

References

[1] A. Amore, R. V. Heerbeek, N. Zeep, J. V. Esch, J. N, H, Reek, H. Hiemstra, J. H. V. Maarsevven, *J. Org. Chem*. 2006, **71**, 1851-1860.

[2] J. H. Park, L. Gu, G. V. Maltzahn, E. Ruoslahti, S. N. Bhatia, M. J. Sailor, *Nat. Materials*, 2009, **8**, 331-336.

[3] S. K. Ko, S. K. Kim, A. Share, V. M. Lynch, J. Park, W. Namkung, W. V. Rossom, N. Busschaert, P. A. Gale, J. L. Sessler, I. Shin, *Nat. Chem*. 2004, **6**, 885-892.
